# Sociodemographic Characteristics as Predictors of Satisfaction in Public and Private Dental Clinics

**DOI:** 10.12669/pjms.345.15519

**Published:** 2018

**Authors:** Aftab Ahmed Khan, Adel Zia Siddiqui, Syed Fareed Mohsin, Badreldin Abdelrhaman Mohamed

**Affiliations:** 1 Aftab Ahmed Khan, MSc, M.Bioeth, B.D.S Researcher, Dental Biomaterials Research Chair, College of Applied Medical Sciences, King Saud University, Riyadh 11433; Saudi Arabia; 2 Adel Zia Siddiqui, MSc, B.D.S Associate Professor, Baqai Dental College, Baqai Medical University, 51 Deh Tor, Toll Plaza, Super Highway, Gadap Road, Karachi 74600; Pakistan; 3 Syed Fareed Mohsin, Ph.D, MSc, MFD RCS, MFDS RCPSG, B.D.S Associate Professor, Department of Oral Pathology/Oral Medicine; College of Dentistry, Qassim University, KSA; 4 Badreldin Abdelrhaman Mohamed, Ph.D, MSc, BSc Professor, Department of Community Health, College of Applied Medical Sciences, King Saud University, Riyadh 11433; Saudi Arabia

**Keywords:** Dental settings, Dental practitioners, Ethnicity, Patients, Satisfaction level

## Abstract

**Objective:**

This study aimed to investigate influence of multiple sociodemographic characteristics on the patient satisfaction levels in outpatient public and private dental practices of Riyadh, Saudi Arabia.

**Methods:**

Questionnaire-based survey data were collected from 500 patients, 250 each from the public and private dental clinics of Riyadh, Saudi Arabia from September to December, 2017. Questions related to demographic factors and service attributes were included. A Likert scale of 5-points was used to measure satisfaction levels. Data was analyzed to calculate the descriptive and inferential statistics (analysis of variance and multiple regression analysis) to find the statistical difference (*p* < 0.01).

**Results:**

Satisfaction level differed significantly by education level (*P*< 0.001) and the type of clinic (*P*<0.001). The multiple regression analysis suggest that all variables influenced satisfaction, except age and marital status. The satisfaction score was higher by 27% for private clinics compared to public clinics.

**Conclusion:**

This study was exploratory and revealed an effect of individual variables on overall satisfaction score of the services attributes. Future plans for patient care could be developed with the help of this research.

## INTRODUCTION

Satisfaction is a complex concept that is difficult to define and measure. It is a psychological term that could be judged over time and experienced by the people.^1^ Simply, it represents the degree to which anticipated goals have been accomplished. Satisfaction encompasses both cognitive and emotional facets and relates to previous experiences, expectations, and social networks.^2^

Donabedian describes four explicit motives for investigating patient satisfaction. First, the objective of care is satisfaction. Second, the consequence of that care is also satisfying, and, therefore, it is an outcome. Third, satisfaction can contribute to the effects of care; a satisfied patient is more presumptive to comply with advice. Finally, satisfaction is also the patient’s judgment of the care that has been provided.^3^

Satisfaction feedback is crucial for continuous enhancement of dental service delivery and promotion of patient–professional relationships.^4^,^5^ These kinds of studies can analyze strengths and weaknesses of health care systems, and the factors influencing patient satisfaction levels can be implemented.^2^ Complaints and dissatisfaction shown by patients may ensue in changing dentist, causing great anxiety and stress among dental care providers.^6^ In an era of clinical governance and delivery of high-quality oral health care, it is necessary to deal with patients’ apprehensions appropriately.^3^, ^7^

Several studies have been conducted to ascertain patients’ satisfaction level in dental-related treatments. In such studies,^1^–^3^ interpersonal factors were the most commonly endorsed reasons among caregivers. Characteristics such as professional competency of the caregiver were much less endorsed. Patients evaluate care quality by their perceptions of caregiver attitudes, conduct and behavior.^8^

Assessing patient satisfaction is vital because it serves as an indicator of overall success in terms of how well an organization is fulfilling the needs of its target populace.^9^, ^10^ Unfortunately, it is easier to define the significance of patient satisfaction than to construct and design suitable instruments to measure it. Different methodologies have been adopted to evaluate patient satisfaction by means of different questionnaires and psychometric tests.^1^ Considering these short falls, our questionnaire was tailored according to the accessible measures in a local dental setting.

There is a growing indication that ethnic minorities have lower satisfaction levels in contrast to majority populations.^11^,^12^ Although plenty of literature is available on determinants and correlates of satisfaction level. However, limited studies have focused on evaluating the variations in satisfaction levels across different ethnic groups, and to our knowledge, ours is the first paper to ascertain the satisfaction level of patients based on multiple sociodemographic factors such as gender, age, civil status, education level and public and private clinical settings to elucidate the associations on ethnicity on patient satisfaction level.

Since Saudi Arabia is a country with high prevalence of dental-related diseases, and large number of expatriates are based in Riyadh. Therefore, we designed our study on determining the factors influencing the satisfaction level on dental patients with different ethnic origin. It was hypothesized that there is no association among the variables on satisfaction level score.

## METHODS

### Study design and participants

This study was approved by the Institutional Review Board for the College of Applied Medical Sciences, King Saud University (IRB 17/23). This observational cross-sectional study was conducted in public and private dental clinics of Riyadh, Saudi Arabia. Participants from the public and private clinics were identified as “practice A” and “practice B” participants, respectively. The respective heads of the institutes gave prior permission and were assured that the names of the institutes would remain confidential. Hence, a total of 250 participants were selected from “practice A”. Similarly, 250 participants were selected from “practice B” for data collection.

### Inclusion criteria

1: Patient 18 years or older.
2: Participants 60 years or younger.
3: Data were collected for participants who had previously visited clinics for general dental treatment.

### Exclusion criteria

1: Participants younger than 18 years or older than 60 years.

### Questionnaire

The questionnaire was designed in English. However, an Arabic interpreter was present all the time for participants only familiar with Arabic language. Questions pertaining to service attributes were designed to effectively evaluate participant satisfaction levels. A Likert scale ranging from 1 to 5 (strongly dissatisfied, dissatisfied, fairly satisfied, satisfied, and strongly satisfied) was used to measure satisfaction level. The questionnaire was coded to maintain privacy and confidentiality of the participants. The first section of a questionnaire was based on sociodemographic data. The second section of the questionnaire included seven questions to judge the overall assessment of satisfaction level (Table-I).

**Table-I:** Mean satisfaction scores according to the socio-demographic characteristics and type of clinical settings of the study groups.

	Number (%) of subjects	Satisfaction score Mean (± SD)	P- value
*Gender*			
Male	265 (53)	3.57 (0.817)	0.109
Female	235 (47)	3.46 (0.717)	
*Marital status*			
Married	389 (77.8)	3.51 (0.711)	0.435
Single	111 (22.2)	3.57 (0.961)	
*Ethnicity*			
Arabs	100 (20)	3.42 (0.632)	
Indians	100 (20)	3.49 (0.573)	0.425
Pakistanis	100 (20)	3.55 (0.602)	
Filipinos	100 (20)	3.62 (0.530)	
Egyptians	100 (20)	3.53 (5.574)	
*Age*			
< 20	6 (1.02)	3.00 (0.560)	
20 - 29	107 (21.4)	3.44 (0.927)	0.067
30 - 39	194 (38.8)	3.50 (0.749)	
40 - 49	113 (22.6)	3.53 (0.714)	
≥ 50	80 (16.0)	3.71 (0.664)	
*Education*			
Illiterate	86 (17.2)	4.402 (0.208)	
Primary	90 (18.0)	4.056 (0.706)	< 0.001
Secondary	109 (21.8)	3.329 (0.558)	
graduate	131 (26.2)	3.068 (0.579)	
Post graduate	84 (16.8)	3.002 (0.578)	
*Type of clinic*			
Practice A	250 (50)	3.350 (0.937)	< 0.001
Practice B	250 (50)	3.691 (0.500)	

SD: standard deviation

### Data collection

Data were collected over a four-month period between September to December, 2017 or until the desired number of questionnaires was completed from each category. A total of 10 dental clinics were targeted (5 for each practice) to collect data (1-2 hour each time) once a week. Each clinic was set to achieve 50 potential participants (10 for each ethnic group). Data were only collected from those participants who had previously visited clinics for general dental treatment. Following informed consent, the required information was collected using the self-answered questionnaire. A member of the study team was available to answer any queries.

### Statistical analysis

The collected data were analyzed with the Statistical Program for Social Sciences (SPSS, Chicago, IL, USA) version 21.0. Descriptive statistics were used to delineate the study population. Analysis of variance was used to estimate the difference in the mean scores of the two types of practices. Finally, a multiple regression analysis was employed to determine the association of various demographic, type of practice, and ethnic factors with the presence of satisfaction levels of dental patients. A *p*-value < 0.05 was considered statistically significant.

## Results

Data were obtained for all the 500 patients selected. The sample was composed of 265 males (53%, mean age = 40.48, ± 9.97) and 235 females (47%, 34.25 ± 9.62). The overall mean age was 37.56 ranging from 18 to 60 years. The majority of the patients (38.8%) were in the age group 30 – 39 years, and most of the patients were married (77.8%). For education, 21.8% reported they were secondary school graduates, and 48% were graduates and post graduates. The overall mean satisfaction score was 3.52 ± 0.773

Mean satisfaction score for services according to socioeconomic variables are shown in Table-I. Analysis of variance showed a significant difference in satisfaction for only education and type of clinic. Regarding age, although no significant difference was detected between age groups, elderly patients showed a higher level of satisfaction than other age groups (3.71). Patients with lower educational levels, illiterate (4.40) and primary (4.06), showed high levels of satisfaction. For type of hospital, patients being treated in practice B showed higher level of satisfaction (3.69) than patients treated in practice A (3.35).

The results of multiple regression of the sociodemographic variables with aspects related to satisfaction with services provided by the dental clinics are presented in Table-II. All variables influenced satisfaction, except age and marital status. Satisfaction is higher by 27% for practice B compared to practice A (unstandardized regression coefficient). For ethnicity, with Arabs as the reference group, all groups included have higher satisfaction than Arabs. Filipinos have the higher increase (29%) in satisfaction of services provided compared to Arabs, while the percentage of increase for other ethnic groups were (13%) for Indians, (17%) for Pakistanis and about 20% for Egyptians. The set of independent variables included in the model account for 55.7% of the variation in satisfaction.

**Table-II:** Predictors of patient satisfaction by multiple regression analysis for services provided.

Predictors	Unstandardized coefficients	Standardized coefficients	P-value
Type of clinic	0.423	0.274	< 0.001
Gender	-0.183	-0.119	< 0.001
Age	0.004	0.051	0.137
Marital status	0.067	0.036	0.265
Education level	-0.406	-0.704	< 0.001
Ethnicity			< 0.001
Indians	0.133	0.069	0.074
Pakistanis	0.166	0.086	0.026
Filipinos	0.285	0.147	< 0.001
Egyptians	0.196	0.120	0.008

**Table-III:** Satisfaction of study group with regard to services provided.

Service item	Strongly dissatisfied	dissatisfied	Fairly satisfied	satisfied	Strongly satisfied	Mean (± SD) (Likert scale)
Reasonable waiting time	41 (8.2)	87 (17.4)	126 (25.2)	159 (31.8)	87 (17.4)	3.33 (1.19)
Attitude of dentist	19 (3.8)	48 (9.6)	154 (30.8)	188 (37.6)	91 (18.2)	3.57 (1.02)
Friendly staff	42 (4.8)	116 (23.2)	146 (29.2)	134 (26.8)	80 (16.0)	3.26 (1.13)
Privacy during treatment	8 (1.6)	25 (5.0)	165 (33.0)	193 (38.6)	109 (21.8)	3.74 (0.91)
Reasonable treating time	34 (6.8)	95 (19.0)	164 (32.8)	104 (20.8)	103 (20.6)	3.29 (1.19)
Cleanliness and neatness of treatment room	2 (0.42)	16 (3.2)	80 (16.0)	216 (43.2)	186 (37.2)	4.14 (0.82)
Convenient appointment time	40 (8.0)	110 (22.0)	123 (24.6)	140 (28.0)	87 (17.4)	3.32 (2.07)

The percentage of satisfied patients was highest for the “cleanliness and neatness of treatment room” (80.4%), “privacy during treatment” (60.4%), “attitude of dentist” (55.8%). Most of the patients were fairly satisfied with all service items, whereas dissatisfaction was recorded lowest for “privacy during treatment (6.6%).

## Discussion

This study is the first to evaluate the influence of multiple sociodemographic characteristics on satisfaction level scores in outpatient public and private dental clinics. These types of studies are necessary to determine patients’ perception of care. Several studies have attempted to discuss patients’ perceptions of satisfaction with care. These include polite and refined behavior of professionals and dental teams.^13^–^15^ This is the only study of its kind to document differences in satisfaction among public and private dental clinic patients, involving several ethnic groups with multiple sociodemographic characteristics. The results could help in revisiting attitudes and behavior of dental professionals and paramedical staff according to the needs and demand of the patients.

Previous studies on patients’ satisfaction have disclosed satisfaction as a complex process with myriad associated factors to unravel.^3^,^16^ Its assessment process has substantial methodological difficulties.^17^ Considering this, we did not try to evaluate questions of service attributes separately. Instead, a short questionnaire with only seven questions related to service attributes was designed, and participants consented to be part of this study without taking too much time. Overall mean satisfaction level score achieved from the service attributes was used to judge the satisfaction level of the participants.

The study’s findings are interesting, in that they partially accept to support our hypothesis. Although, the variables such as gender, marital status, age and ethnicity have comparable levels of satisfaction without statistical differences, and in accordance with the previous findings.^18^-^20^ However, education and type of clinic found to have influence on the mean satisfaction score of the participants.

The findings of this study suggest statistical difference among the participants with different education level which is, in fact, not surprising (*P*=< 0.001). A person with higher education level might have a capacity to perceive and understand the working situation at the clinics in a better way.^21^,^22^ On the contrary, those with lower education level might not be aware of the reception and care they are entitled to, and hence due to lower education level or illiteracy, they are deprived of the important indicator to judge the socioeconomic level in a society.^21^, ^22^ This study also revealed a significant difference between the two types of (*P*=< 0.001).

By contrast, dentists and paramedical staff in private clinics are well-trained and experienced. They usually switch their jobs to private clinics due to increased monetary benefits.^23^ Whereas, the dentists and paramedical staff in public clinics are relatively new to field. In addition, the work load in public clinics are higher compared to private clinics where appointment-based system is strictly followed to see the patients. This allows doctors in private clinics to spend more time with patients, and patients are generally more satisfied with the experience, especially when it comes to them feeling like all of their concerns have been addressed. The interpersonal factors of public clinic characteristics could be the dominant reasons behind this difference. The work load and lower staff-to-patient ratio could also explain the lower satisfaction level of the practice A participants.

We used multiple regression analysis to model how a large number of factors influence overall satisfaction score and their relative influences. Through multiple regression, it is possible to identify the factors that do not have statistically significant effect. Our findings suggest that age (*P*=0.137) and marital status (*P*=0.265) have no influence on the overall satisfaction score.

Overall, it is seen that the patients of both clinics, i.e., practice A (score=3.35±0.93) and practice B (score=3.691±0.50) did not rate the satisfaction score higher for the working staff. It is strongly suggested that the working staff should constantly be smiling at the patients. Smiling will make the nervous and worried patients calm and easy.^24^ Moreover, the mean satisfaction score of both clinics was also rated low in score (3.29±1.19). Time spent on treating a patient should not be long since it may annoy and frustrate the patient, and the patient might be unwilling to return outpatients dental settings.^25^

With knowledge and technical skills, good dental treatment is possible. With effective two-way communication, ethical consideration, professionalism, and patient satisfaction could be attained. This study strongly suggests the importance of service improvement and communication in public practices. Better-educated dental practitioners have better attitudes towards providing care, leading to higher patient satisfaction, a vital characteristic for analyzing overall quality of care.^1^

### Strength and Limitations of the study

The strength of this study lies in the larger sample size of different ethnic origin which may justify to generalize the findings. However, limitation of this study was provider factors, i.e., caregiver gender. Female doctors use a more patient-centered approach than their male counterparts, which greatly affects patient satisfaction. Since the dentists of “practice A” were male only whereas “practice B” had both male and female dentists. This could have affected the satisfaction score. Furthermore, the study population was selected from urban area only. The public clinic participants belonged to low socioeconomic strata comparably with lower education level; the exclusion of participants from other socioeconomic strata might create biasness. Nevertheless, the perception of the majority could be assessed with the available data. For future studies, it is recommended to select a stratified sampling method to clarify any ambiguity.

## Conclusion

This study attempted to compare the satisfaction level of the patients in public and private clinics of Riyadh, Saudi Arabia. Several ethnic groups with multiple sociodemographic characteristics were considered in the study. The study showed that variables such as gender, age, civil status and ethnicity do not significantly impact upon a patient’s satisfaction. However, substantial disparity was found in education and both types of clinical settings. Although the overall satisfaction score was high, however some areas were highlighted where improvements could be made. This study was exploratory and revealing for all public dental clinics of Riyadh, Saudi Arabia. The information gathered from this study could be useful in developing future plans of the public clinics.

### Authors’ Contribution

**AAK:** Designed the study, data collection, wrote methodology and discussion.

**AZS:** Contributed in writing introduction and final editing of references.

**SFM:** Did critical review and final editing of this write up.

**BAM:** Did statistical analysis and results interpretation.

## References

[1] Sun N, Burnside G, Harris R (2010). Patient satisfaction with care by dental therapists. Br Dent J.

[2] Armfield JM, Enkling N, Wolf CA, Ramseier CA (2014). Dental fear and satisfaction with dental services in Switzerland. J Public Health Dent.

[3] Sur H, Hayran O, Yildirim C, Mumcu G (2004). Patient satisfaction in dental outpatient clinics in Turkey. Croat Med J.

[4] Othman N, Razak IA (2010). Satisfaction with school dental service provided by mobile dental squads. Asia Pac J Public Health.

[5] Kamimura A, Ashby J, Myers K, Nourian MM, Christensen N (2015). Satisfaction with healthcare services among free clinic patients. J Community Health.

[6] Crossley ML, Blinkhorn A, Cox M (2001). What do our patients really want from us?Investigating patients'perceptions of the validity of the Charter mark criteria. Br Dent J.

[7] Suki NM (2011). Assessing patient satisfaction, trust, commitment, loyalty and doctors'reputation towards doctor services. Pak J Med Sci.

[8] Mataki S (2000). Patient–dentist relationship. J Med Dent Sci.

[9] Mascarenhas AK (2001). Patient satisfaction with the comprehensive care model of dental care delivery. J Dent Educ.

[10] Hajifathali A, Ainy E, Jafari H, Moghadam NM, Kohyar E, Hajikaram S (2008). In-patient satisfaction and its related factors in Taleghani University Hospital, Tehran, Iran. Pak J Med Sci.

[11] Kirmanoglu H, Baslevent C (2014). Life satisfaction of ethnic minority members:an examination of interactions with immigration, discrimination, and citizenship. Soc Indi Res.

[12] Bobowik M, Basabe N, Paez D (2015). The bright side of migration:hedonic, psychological, and social well-being in immigrants in Spain. Soc Sci Res.

[13] Fox C (2010). Evidence summary:what do we know from qualitative research about people's care-seeking about oral health?. Br Dent J.

[14] Anderson R (2004). Patient expectations of emergency dental services:a qualitative interview study. Br Dent J.

[15] Brennan DS, Spencer AJ (2006). Dentist preferences for patients:dimensions and associations with provider, practice, and service characteristics. Int J Behav Med.

[16] Newsome PR, Wright GH (1999). A review of patient satisfaction:2. Dental patient satisfaction:an appraisal of recent literature. Br Dent J.

[17] Sitzia J, Wood N (1997). Patient satisfaction:a review of issues and concepts. Soc. Sci. Med.

[18] Weech-Maldonado R, Morales LS, Spritzer K, Elliott M, Hays RD (2001). Racial and ethnic differences in parents'assessments of pediatric care in Medicaid managed care. Health Serv Res.

[19] Weech-Maldonado R, Elliott MN, Morales LS, Spritzer K, Marshall GN, Hays RD (2004). Health Plan Effects on Patient Assessments of Medicaid Managed Care Among Racial/Ethnic Minorities. J Gen Intern Med.

[20] Kim M, Zaslavsky AM, Cleary PD (2005). Adjusting pediatric Consumer Assessment of Health Plans Study (CAHPS) scores to ensure fair comparison of health plan performances. Med Care.

[21] Barlesi F, Boyer L, Doddoli C, Antoniotti S, Thomas P, Auquier P (2005). The Place of Patient Satisfaction in Quality Assessment of Lung Cancer Thoracic Surgery. Chest.

[22] Fiscella K, Goodwin MA, Stange KC (2002). Does patient educational level affect office visits to family physicians?. J Natl. Med. Assoc.

[23] Lo Sasso AT, Starkel RL, Warren MN, Guay AH, Vujicic M (2015). Practice settings and dentists'job satisfaction. J Amer Dent Assoc.

[24] Ayyub R, Kanji Z, Dias J, Roshan R (2015). Perceptions of Patients Regarding Quality Nursing Care (QNC) at a Tertiary Care Hospital, Karachi, Pakistan. J Clin Res Bioeth.

[25] Daniels J, Zweigenthal V, Reagon G (2017). Assessing the impact of a waiting time survey on reducing waiting times in urban primary care clinics in Cape Town, South Africa. J Public Health Afr.

